# Intersectionality-based quantitative health research and sex/gender sensitivity: a scoping review

**DOI:** 10.1186/s12939-019-1098-8

**Published:** 2019-12-21

**Authors:** Emily Mena, Gabriele Bolte, on behalf of the ADVANCE GENDER Study Group, Gabriele Bolte, Emily Mena, Alexander Rommel, Anke-Christine Saß, Kathleen Pöge, Sarah Strasser, Christine Holmberg, Sibille Merz, Philipp Jaehn

**Affiliations:** 1 Institute of Public Health and Nursing Research, Department of Social Epidemiology, Faculty of Human and Health Sciences, University of Bremen, Grazer Straße 4, 28359 Bremen, Germany; 2 Health Sciences Bremen, University of Bremen, Bremen, Germany

**Keywords:** Epidemiology, Health equity, Intersectionality, Sex/gender, Diabetes, Smoking, Physical activity, Health reporting

## Abstract

**Background:**

The implementation of a theoretical intersectionality framework into quantitative data analyses is gaining increasing interest in health research. The substantive foundation of intersectionality was established in the U.S., based on the claim of black feminists to broaden the scope of contemporary gender studies by considering the intersection between sex/gender and race/ethnicity more firmly. The aim of our scoping review with particular emphasis on sex/gender was to assess how intersectionality-informed studies in epidemiological research considered different social dimensions in their multivariable and multivariate analyses.

**Methods:**

Following the PRISMA Extension for Scoping Reviews (PRISMA-ScR), we conducted a literature review in PubMed. Three distinct health-related fields were brought into focus: diabetes representing a frequent chronic disease, smoking as a wide-spread behavioural health determinant and physical activity as a central target for health promotion. Initially, we compared which and how different social dimensions were accounted for and how inter-categorical and intersectionality-informed analyses were conducted. Further, we assessed sex/gender sensitivity by comparing operationalisation of sex/gender, how sex/gender theories were used and which central theoretical sex/gender concepts were referred to when aiming at explanation of (intersectional) sex/gender differences.

**Results:**

Our results suggest, that intersectionality-based analyses within the three selected health-related fields are mainly conducted in the U.S. and focused on the intersection between sex/gender and race/ethnicity by using them jointly as subgrouping variables and as parts of interaction terms in regression analyses. Income and education as proxies for social class as well as age are mainly used for adjustment in quantitative analyses. Other approaches for calculating interactions (i.a. synergy-index, CART-analysis) are an exception. Even though sex/gender was considered in every included study and Gender was the most frequent theoretical sex/gender concept referred to when theoretically explaining sex/gender differences, it was exclusively operationalised as binary and solution-linked sex/gender variables were hardly considered in quantitative analyses.

**Conclusion:**

The systematic integration of solution-linked variables indicating modifiable aspects of sex/gender-related living conditions and disadvantages could improve sex/gender sensitivity as part of intersectionality-based quantitative data analysis in health research.

## Key points

- Sex/gender as main category of all intersectionality-informed studies
- Sex/gender always operationalised binary despite multidimensional gender concept
- Focus of all studies from the U.S. on interaction with race/ethnicity
- Stratification or interaction terms in regression models as main analyses strategy
- Neglect of solution-linked, modifiable sex/gender aspects

## Background

It has been suggested that understanding of complex causes and mechanisms leading to health inequalities will be improved by integration of an intersectionality framework into health research [1]. To date, intersectionality theory has only been extensively considered in qualitative health research [1–4]. In contrast, discussion on how intersectionality theory could be implemented in quantitative health research just started during the past years [1, 5]. In this regard, Seng et al. [3] proposed to operationalize intersectionality from an ecosocial perspective and to model demographic characteristics across different levels capturing the macro-, exo-, meso- and micro-system. A most recent development, although criticized [6], is based on Merlo’s [7] multilevel analytical approach in social epidemiology, by expanding multilevel analysis of individual heterogeneity and discriminatory accuracy (MAIHDA) into an intersectional framework [8–10]. Moreover, in order to reduce health inequities, the consideration of modifiable societal and contextual factors is increasingly called upon [11, 12].

Against the background of the fundamental impact of sex/gender on health [13–15] and the debate among intersectionality scholars, whether gender should be the starting point of theory and analysis [16, 17] or not [4, 18], Hammarström et al. [19] recommended to study dynamics of sex/gender, complex intersections, social context, and power relations. Health monitoring and reporting as one important source for evidence-based policy making relies on valid epidemiological research [20]. Consideration of sex/gender at least as binary individual characteristic is nowadays a standard approach in health reporting [21]. Usage of statistical methods from an intersectionality perspective to assess more comprehensively interrelations and dynamics at several levels in epidemiological health research could further improve health reporting and its sex/gender sensitivity.

The implementation of an intersectionality framework into quantitative health research offers the possibility to further intercategorical analyses [22] and to explore a variety of possibly interacting social dimensions. However, the theoretical concept of intersectionality originated from feminist scholarship [19], but it is not clear, if the focus on sex/gender is currently part of the implementation of intersectionality into quantitative data analyses as well. Efforts to implement intersectionality into quantitative health research might differ with regard to sex/gender being considered as a master category or not, in terms of theoretical embeddedness of the research question and results as well as regarding the choice of modelling strategy. Therefore, the aim of our scoping review was to assess whether and how recent studies, conducted by authors that explicitly refer to intersectionality, operationalized and considered socio-cultural, socio-economic, and demographic aspects, quantitatively analysed interactions, and integrated gender theoretical concepts and explanations. Within three selected thematic fields relevant for health reporting, the following case studies were chosen: diabetes as a frequent chronic disease, smoking as one of the most important behavioural health determinants, and physical activity as one of the most relevant targets for health promotion.

## Methods

This scoping review was carried out following the PRISMA Extension for Scoping Reviews - PRISMA-ScR [23] within the research project AdvanceDataAnalysis. This project is part of the collaborative research project AdvanceGender [21] and aims to promote sex/gender-sensitive and intersectional quantitative health research and health reporting.

### Search strategy

The PubMed database was searched on May 14, 2019 using the following three search strategies with no restrictions regarding language or publication date:

Diabetes:

(intersect*[Title/Abstract]) AND (diabet*[Title] OR “metabolic syndrome”[Title])

Smoking:

(intersect*[Title/Abstract]) AND (smok*[Title] OR tobacco [Title])

Physical activity:

(intersect*[Title/Abstract]) AND (physical activi*[Title] OR exercis*[Title] OR sport*[Title] OR walk*[Title] OR active commut*[Title] OR sedentary behavio*[Title] OR physical inactivi*[Title] OR cycling [Title])

### Study selection and inclusion criteria

We included studies on adults within the three defined thematic fields, which conducted quantitative data analysis and referred to intersectionality at least in title or abstract. There was no restriction of the research question. Studies which did not explicitly mention intersectionality but nevertheless used intersectionality terminology, especially using terms like “intersection” for the combination of two demographic, socio-cultural and/or socio-economic variables (in the following termed intersectional-variables; for more details on defined variables see Table 1), were additionally regarded as intersectionality-based and included into the review.

**Table 1:**
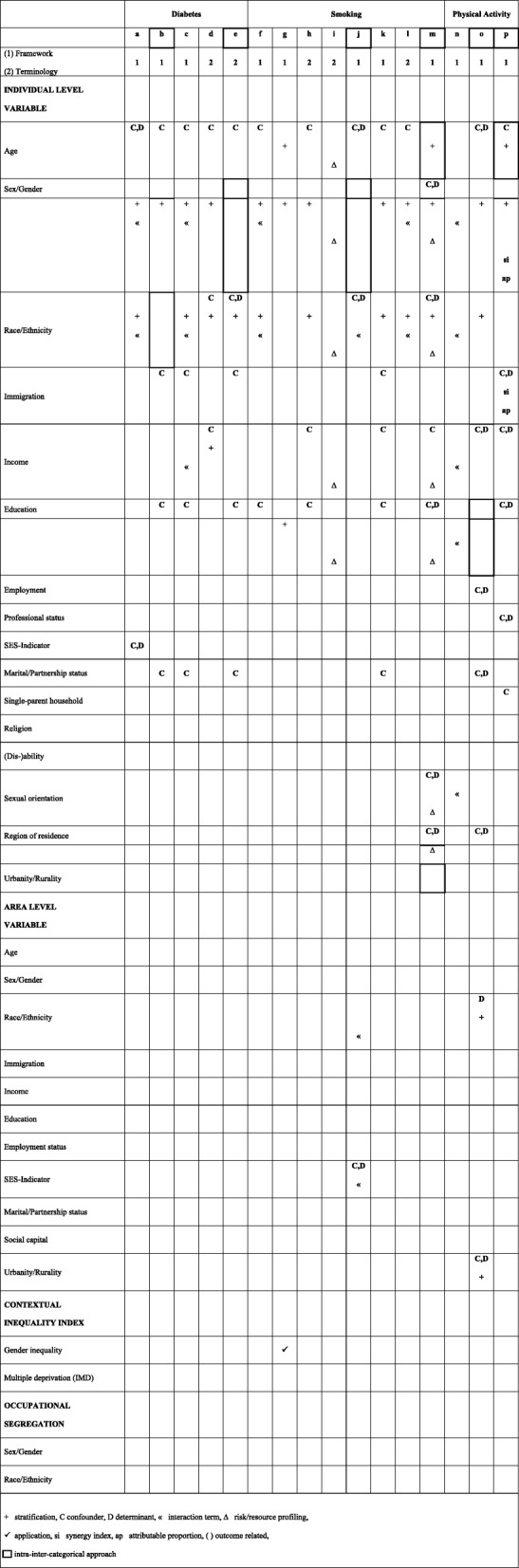
Intersectionality theoretical framework or terminology and definition and function of intersectional variables in multivariable analysis

Title/abstract screening was performed by two reviewers independently, any disagreements were solved by discussion with a third reviewer. Following the title/abstract screening, we included only full-text articles which reported results on any kind of analyses like interaction terms in regression analyses, stratified models or risk/resource profiles.

Included studies not investigating a particular population subsample a priori were defined as analyses with an inter-categorical approach. McCall [22] defines inter-categorical complexity as a comparative and multigroup approach in intersectionality related research, which uses categorization strategically and is suitable for application of quantitative methods. For better comparability we defined studies restricted to a population subsample characterized by at least one intersectional-variable from the start as analyses with an intra-inter-categorical approach. Full-text articles with an inter-categorical approach were not excluded if at least two intersectional-variables for modelling interaction were considered and multivariable or multivariate analyses [24] were utilised as main methodological procedure. Articles applying an intra-inter-categorical approach were not excluded if at least one other intersectional-variable was considered in multivariable or multivariate analyses (hereinafter referred to as multivariable analyses). Since analyses with an intra-inter-categorical approach were restricted to a certain (intersectional) population subsample a priori, interaction analyses were defined from a theoretical perspective, therefore studies conducting main effect analyses with an intra-inter-categorical approach were not excluded.

### Data extraction

Data was extracted by one reviewer and completely checked by the second reviewer. Any disagreements were solved by discussion. Data extracted from full-text included first authors, publication year, title, study location, database, study population, research design, inter-categorical or intra-inter-categorical approach and main methodological multivariable analyses.

First, we assessed and rated how analyses were embedded in intersectionality theoretical framework (TFW) (1 = Intersectionality TFW is stated as theoretical background for conducting analysis or mentioned and discussed somewhere throughout the paper; 2 = Intersectionality TFW terminology is used, without specifically referring to an intersectionality TFW).

Second, we compared the different statistical strategies applied when modelling interaction by assessing the use and function of the different intersectional-variables when calculating effect estimates. For this purpose, relying on the Progress-Plus Framework [25] we defined intersectional-variables at the individual level (age, sex/gender, race/ethnicity, income, education, employment status, professional status, socio-economic status/class-indicator (SES-indicator), marital/partnership status, children, single-parent household, immigration, religion, dis/ability, sexual orientation, region of residence, urbanity/rurality) and area level (age, sex/gender, race/ethnicity, immigration, income, education, employment status, SES-indicator, marital/partnership status, social capital, urbanity/rurality), and regarding contextual inequality indices (gender inequality, indices of multiple deprivation) as well as occupational segregation (sex/gender, race/ethnicity). Potential use and functions of intersectional-variables within multivariable analyses were confounder (in part without reporting effect estimates), independent determinant for the analysed outcome (e.g. mutually adjustment for several independent variables), stratification, being part of an interaction term, use for risk/resource factor profiling (e.g. CART-analysis, MAIHDA), and modelling as intermediate variable. Moreover, results of interaction analyses at the additive scale might be reported as synergy index, RERI (relative excess risk due to interaction) or attributable proportion.

Third, we assessed operationalisation of sex/gender and available information about respective data collection as well as any other additional information regarding operationalisation of sex/gender.

Fourth, we defined criteria for assessment of gender sensitivity following a “solution-based” approach, which incorporates modifiable societal and contextual factors accountable for marginalization of socially defined groups due to unequal power relations [11]. Correspondingly, “solution-linked variables”, which are variables that actually drive heterogeneity across social dimensions [1, 11] and therefore are relevant for explaining sex/gender differences, were defined. Relying on a gender concept, that to a large extent was derived from the Canadian Institutes of Health Research [26] and was used to develop a composite measure of gender [27], we defined the following solution-linked variables: employment status, education, personal income, family constellation, financial responsibilities, care responsibilities, housework responsibilities, stress level or management, and social support or conflict. Discrimination was additionally defined as a solution-linked variable since it is an important mediating process regarding outcome inequalities from an intersectionality perspective [28, 29]. We compared if and how these solution-linked variables were accounted for in the models by comparing their use and function in the included studies, as done with the intersectional-variables. In addition, we checked if solution-linked variables were presented descriptively.

Fifth, relying on Hammarström and Hensing’s [30] investigation on how gender theories are used in contemporary public health research, we assessed whether gender theories were used to test hypotheses, to be integrated in various parts of the paper, to develop gender concept and models, to interpret empirical findings, to understand health problems, to illustrate the validity of theories with health status or health-related behaviour as example, to be integrated in traditional gender blind theories and/or to criticise other feminist theories.

Sixth, sex/gender sensitivity was assessed by identifying text passages aiming at explaining sex/gender differences and assigning the text passages to respective central theoretical sex/gender concepts [19] (see Additional file 1). Our understanding of sex/gender differences within an intersectionality framework included comparisons between men and women, but also comparisons of subgroups characterized by any further intersectional variable within one gender group, comparison e.g. of black women vs white women, white men or black men. Accordingly, we did not define intersectionality as one of the central theoretical sex/gender concepts defined by Hammarström et al. [19], but as an overarching analytical frame applicable to the other identified central theoretical sex/gender concepts in health research: Gender, Gender Equality, Gender Equity, Embodiment and Sex. We further differentiated if explanations referring to the theoretical concept Gender described intra-individual and/or inter-individual processes and sorted the extracted text passages into thematic categories. The theoretical concept Embodiment was divided into three conceptualisations summarised by Hammarström et al. [19]: The epidemiological perspective describes bodily changes due to the material and social world, thus constituting different population patterns of health and disease. Social embodiment focuses on the interrelationship between bodies, social relations and social structure as a collective and reflexive process. Phenomenology relates to the “lived body” and the mind-body-world as an inseparably interwoven entirety.

## Results

The search strategies led to the identification of 484 possibly relevant articles (diabetes = 141; smoking = 80; physical activity = 263). Excluded studies mostly did not refer to intersectionality (*n* = 438) or did not conduct multivariable analysis (*n* = 25) (see Additional file 2). Overall 21 articles met our inclusion criteria at the title/abstract level (diabetes = 6; smoking = 11; physical activity = 4). All studies were published in English. Exclusion of publications not mainly focusing on the adult population (four studies) or not conducting interaction analyses (e.g. interaction terms in regression analyses, stratified models, risk/resource profiles) (one study) resulted in a total of 16 included studies (diabetes = 5; smoking = 8; physical activity = 3). For bibliography data see Additional file 3. Of the 16 articles meeting eligibility criteria (from now on referred to with the letters a-p), 12 were conducted in the U.S. (a,d,e,f,g,h,i,j,k,l,m,o), two in Canada (c,n), one in Spain (b) and one in Sweden (p). Ten out of the 16 included studies followed an inter-categorical approach (a,c,d,f,g,h,i,k,l,n,), six an intra-inter-categorical approach. Intra-inter-categorical approaches focused at baseline on women (e,j), on young adults (m,p), on immigrants (b) or on participants holding at least a Bachelor’s degree and living in (sub-)urban areas (o). With the exception of two included studies (c,f,) all other studies reported regression-based methods as main methodological approach in multivariable analyses (a,b,d,e,g,h,i,j,k,l,m,n,o,p) (see Additional file 4).

Table 1 shows whether the studies used only certain terminology (e.g. using the word intersect in combination with at least two intersectional variables) or referred to intersectionality theoretical framework. Furthermore, it gives an overview with respect to the function of intersectional variables in multivariable analyses. Five studies referred to an intersectionality framework at the title/abstract level (a,c,j,n,o), all other studies used only intersectionality related terminology (b,d,e,f,g,h,i,k,l,m,p) (information on title/abstract not shown in Table 1). At the full-text level overall 11 articles referred to intersectionality as theoretical framework for conducting analyses (a,b,c,f,g,j,k,m,n,o,p) and five studies continued using only intersectionality related terminology (d,e,h,i,l) (Table 1). Age was included in most studies (a,b,c,d,e,f,g,h,i,j,k,l,m,o,p), mainly as confounder. Sex/gender was considered in every included publication. Race/ethnicity was addressed in most studies (a,b,c,d,e,f,h,i,j,k,l,m,n,o). Sex/gender and race/ethnicity were predominantly used for stratification. Sex/gender and race/ethnicity were primarily used jointly as subgrouping variables (a,b,c,d,e,f,h,k,l,m,o) and parts of interaction terms (a,c,f,l,n). Other intersectional-variables like education (b,c,e,f,h,k,m,p), marital/partnership status (b,c,e,k,o), income (d,h,k,m,o,p) or immigration (b,c,e,k,p) were considered in about half or less of the included studies, for the most part as confounders. Employment (o), professional status (p) SES-indicator (a) and single-parent household (p) were rarely used in multivariable analyses. If applied, they were used in mutually adjusted models (a,e,j,m,o,p). Area-level intersectional variables(j,o) and contextual inequality indices (g) were infrequently considered. Intersectional variables regarding occupational segregation variables were not regarded at all. Measures of interaction at the additive scale such as synergy index and attributable proportion were applied in one paper on interactions between sex/gender and immigration (p). Risk/resource profiling was utilized with two studies conducting CART-Analysis (i,m). Conclusions regarding differences in use and function of intersectional-variables when comparing inter-categorical or intra-inter-categorical approaches cannot be drawn. None of the included studies modelled intersectional-variables as intermediate factors.

Table 2 presents operationalisations of sex/gender and gives insight about information provided by the authors regarding sex/gender data collection. All 14 studies, that were not following an intra-inter-categorical approach regarding one sex/gender (a,b,c,d,f,g,h,i,k,l,m,n,o,p), operationalised sex/gender consistently as binary (men vs women). The two studies investigating only women (e,j), did also not further categorize sex/gender (e.g. different categories of sex/gender identity) and thus relied on the binary view, too. More than half of the included studies did not specify how data about sex/gender was gathered (b,c,d,g,i,k,n,o,p). Two studies used and reported information about sex/gender retrieved from register data (e,j), five studies provided information about sex/gender being self-reported or self-identified by the participants (a,f,h,l,m), and two studies indicated forced choice (f,l).

**Table 2:**
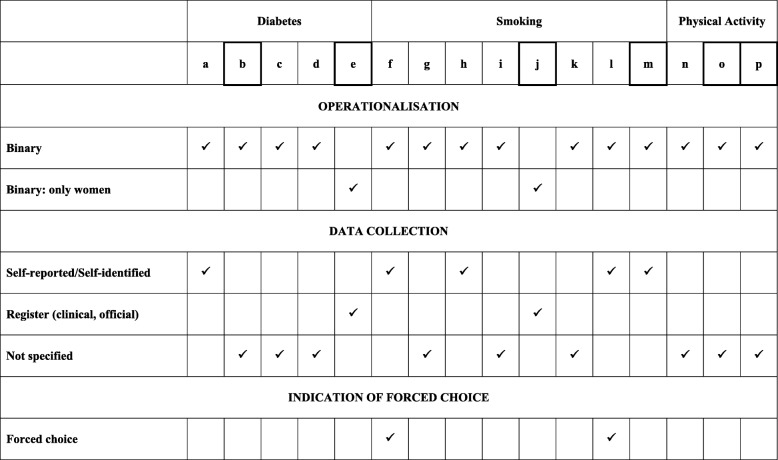
Operationalisation of sex/gender and information about data collection

Table 3 shows the use of solution-linked sex/gender variables. None of the included studies considered further solution-linked variables, that were not already reported with respect to intersectional variables (employment, education, family constellation). The respective variables were all presented descriptively. Most of the studies referring to family constellation used marital/partnership status (b,c,e,k,o), one article referred to children living in the home (o) and one study addressed dual- or single-parent household (p) to operationalise family constellation. Regarding all other solution-linked variables that were not concurrently conceptualized as intersectional-variables, only one study provided additional descriptive information about outcome-related distress and social support (b). Comparing inter-categorical or intra-inter-categorical approaches, no conclusions regarding differences in operationalisation, information provided about data collection and the use of solution-linked sex/gender variables can be drawn.

**Table 3:**
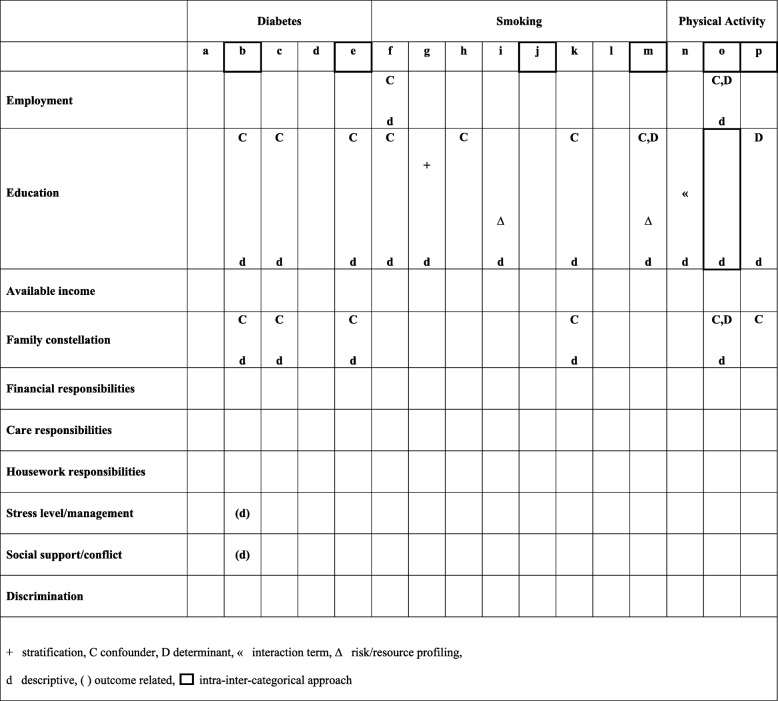
Use of solution-linked sex/gender variables

Table 4 shows how sex/gender theories where used in the articles. In five out of the 16 included studies (e,h,i,k,m) none of the pre-defined strategies for the use of sex/gender theories were applied. Only four out of overall eight defined strategies were used in the included publications. The strategies applied were to interpret empirical findings (a,b,c,d,f,g,l,n,o,p), understand health problems (a,b,c,d,f,g,j,l,o,p), integrate gender theories in various parts of the paper (a,b,f,g,j,l,no,p), and to test hypotheses (a,b,f,g,l,o). The amount of strategies for use of sex/gender theories in the studies varied between 2 and 4.

**Table 4:** Use of sex/gender theories in intersectionality-based quantitative analyses

Sex/gender theories were used to																
	Diabetes					Smoking								Physical Activity		
	a	b	c	d	e	f	g	h	i	j	k	l	m	n	o	p
test hypotheses	**✓**	**✓**				**✓**	**✓**					**✓**			**✓**	
be integrated in various parts of the paper	**✓**	**✓**				**✓**	**✓**			**✓**		**✓**		**✓**	**✓**	**✓**
to develop gender concept and models																
interpret empirical findings	**✓**	**✓**	**✓**	**✓**		**✓**	**✓**					**✓**		**✓**	**✓**	**✓**
understand health problems	**✓**	**✓**	**✓**	**✓**		**✓**	**✓**			**✓**		**✓**			**✓**	**✓**
to illustrate the validity of theories with health status as example																
to be integrated in traditional gender blind theories																
to criticise other feminist theories																

Table 5 provides information about which central sex/gender theoretical concepts are referred to when aiming at explanation of intersectional sex/gender differences and specifies thematically which intra- and inter-individual processes are considered when relying on Gender as a theoretical concept. For overall four out of the 16 included publications, we could not extract a single text passage aiming at explanation of sex/gender differences. Eleven out of the remaining 12 articles used Gender as a central theoretical concept (a,b,c,d,f,g,j,l,n,o,p). Less than half of the included studies providing explanations for sex/gender differences referred to the other central gender theoretical concepts Gender Equality (b,e,g,o,p), Gender Equity (b,e,g,o), Embodiment (f,g,j,l) and/or Sex (d,e,l). The number of central gender theoretical concepts addressed within one paper varied between 1 and 4. Regarding the further classification of the theoretical concept Gender into intra- and inter-individual processes, we found psychological/cognitive (a,b,f,l,o), physical/biological (j,l), behavioural (f,j,p) and privilege/resource-related (c) aspects as intra-individual processes referred to, when explaining sex/gender differences. Inter-individual processes within the theoretical concept Gender were gender norms (b,c,d,f,g,n,o,p), socio-cultural (a,b,f,g,j,l,o), discrimination (b,c,d,o,p), social networks/social capital-related (a,b,o), privilege/resource-related (j,n), socio-economic (n,p) and environmental factors (j). Comparing inter-categorical or intra-inter-categorical approaches, no conclusions regarding differences in use of sex/gender theories and explanations of intersectional sex/gender differences can be drawn.

**Table 5:** Use of central sex/gender theoretical concepts and processes for explanation of intersectional sex/gender differences

Sex/gender concepts and processes for explanation of intersectional sex/gender differences																
	Diabetes					Smoking								Physical Activity		
	a	b	c	d	e	f	g	h	i	j	k	l	m	n	o	p
Gender (intra-individual)	**✓**	**✓**	**✓**			**✓**				**✓**		**✓**			**✓**	**✓**
*Psychological/cognitive*	**✓**	**✓**				**✓**						**✓**			**✓**	
*Physical/biological*										**✓**		**✓**				
*Behavioural*						**✓**				**✓**						**✓**
*Privilege/resource*			**✓**													
Gender (inter-individual)	**✓**	**✓**	**✓**	**✓**		**✓**	**✓**			**✓**		**✓**		**✓**	**✓**	**✓**
*Gender norms*		**✓**	**✓**	**✓**		**✓**	**✓**							**✓**	**✓**	**✓**
*Socio-cultural*	**✓**	**✓**				**✓**	**✓**			**✓**		**✓**			**✓**	
*Discrimination*		**✓**	**✓**	**✓**											**✓**	**✓**
*Social networks/social capital*	**✓**	**✓**													**✓**	
*Privilege/resource*										**✓**				**✓**		
*Socio-economic*														**✓**		**✓**
*Environmental*										**✓**						
Gender Equality		**✓**			**✓**		**✓**								**✓**	**✓**
Gender Equity		**✓**			**✓**		**✓**								**✓**	
EMBODIMENT						**✓**	**✓**			**✓**		**✓**				
*Eco-social*										**✓**						
*Social (behavioural)*						**✓**	**✓**			**✓**						
*Psycho-biological*												**✓**				
Sex				**✓**	**✓**							**✓**				

## Discussion

The objective of our scoping review was to assess how intersectionality-based approaches are realized in quantitative data analyses and how sex/gender sensitivity is accounted for. The majority of the studies is based on data retrieved from the U.S. American population. Sex/gender was the only intersectional-variable used in every study without exception. None of these studies considered other sex/gender dimensions beyond male or female, e.g. gender identity. Information about how data about sex/gender was retrieved were rarely provided. None of the included studies integrated available income, financial responsibilities, care responsibilities, housework responsibilities, stress level/management, social support/conflict or discrimination as solution-linked sex/gender variables into description or analysis. The most common methodological approach for analysis of interaction at the multiplicative scale were regression-based methods, mainly with stratified analysis and/or use of interaction terms. Both strategies primarily considered sex/gender and race/ethnicity jointly as intersectional-variables. Most studies applying interaction terms referred to intersectionality as a theoretical framework and used at least between 2 and 4 of the defined strategies for applying gender theories. Our results regarding the use of strategies for applying gender theories are in line with Hammarström and Hensing [30], who found the same four out of eight strategies used in health research studies conducting quantitative analyses.

Bowleg [31] advises intersectionality scholars to use the word intersectionality in titles, keywords, abstracts, or articles to further a coherent development in this research area. Regarding the included studies applying interaction terms in regression models, all studies without exception referred to intersectionality as a theoretical framework. This might reflect a tendency towards a more profound examination of the research question within an intersectionality framework, when interaction terms in regression models are considered as main methodological approach.

Many aspects of analysis strategies based on an intersectionality framework are already part of modern social epidemiology [32–34]. Thus, by focusing on the term intersectionality in this review other relevant studies might have been missed. It would be worthwhile to further analyse how intersectionality-based studies and modern social epidemiological studies, respectively, use interaction analyses and other more sophisticated methods to investigate e.g. processes of discrimination and power relations as causes of health inequalities in contrast to studies relying on the risk factor paradigm in conventional epidemiology [35–37].

The high proportion of studies referring to intersectionality and thereby primarily focusing on the intersection between sex/gender and race/ethnicity seems best explained by the origin of intersectionality, which is rooted in black feminism [38]. The very frequent consideration of race/ethnicity as intersectional-variable in combination with sex/gender might be a consequence of “the false notion that hierarchical racial categories reflect (ed) biological realities” [39], which is particularly pronounced in the U.S. as part of its historical development. As a result, the objectively unjustifiable categorization, demarcation and discrimination of citizens due to unbalanced power relations and power exercise [38] may have led to the popularity of the intersectionality concept in the U.S., including the focus on the intersection between sex/gender and race/ethnicity. Furthermore, differences regarding available data in light of the history of race/ethnicity measurement in various countries, e.g. in a European context [40], might be another reason for researchers outside the U.S. not to focus explicitly on the intersection between sex/gender and race/ethnicity. The only studies that did not model race/ethnicity as intersectional-variable were two out of overall four studies conducted outside of the U.S. and are thus broadening the scope of the intersectionality framework towards the consideration of different intersectional-variables simultaneously. Of note, theories like e.g. minority stress theory [41], that could be suitable for theorising intersectionality from a simultaneous perspective, i.e. not preferring one intersectional-variable over another a priori [4], were rarely mentioned in the studies of this review. Only few papers referred to Multiple Jeopardy (c,n) [42], Theories of social stratification (j) or Acute social invisibility (n) [43], terms that might be linked to intersectionality-related theories from a simultaneous perspective. With view on intersectionality from a simultaneous perspective, we could not find an equivalent to the review of central theoretical sex/gender concepts in health research by Hammarström et al. [19], who aimed at contributing to greater conceptual stringency and provision of a sound conceptualisation for gender research in health sciences. A review of theories linked to intersectionality from a simultaneous perspective might enhance the implementation of intersectionality into population health research – with respect to theory and methodology.

Even though our results can only be interpreted cautiously, it does stand out that the two studies conducting CART-Analysis (i,m) are two out of five studies, that did not refer to any gender theories (e,h,i,k,m) or any other intersectionality-related theory. In addition, both studies used almost none of the defined solution-linked sex/gender variables. “Although theory development is relatively uncommon in the health sciences compared with other disciplines, it is vital to have clear and well-developed concepts in order to develop well-specified and appropriate research questions” [19]. However, CART-Analysis is a non-parametric procedure which allows for simultaneous consideration of multiple intersectional-variables and makes no assumptions about data distribution or independence [44]. Therefore, from a methodological perspective, CART-Analysis might be suitable for statistical modelling of the concept of intersectionality by considering different intersectional-variables simultaneously.

The majority of included studies are based on data analyses of national surveys. In contrast, only one relevant intervention study was found. This finding is not surprising since interaction analyses in general rely on data retrieved from large surveys that can provide sufficient statistical power. In turn, the choice of a modelling strategy could be impacted as well. A key lever for implementation of intersectionality-informed studies would be standardized data collection, especially with focus on national surveys, that allows to capture a wide variety of socio-cultural, socio-economic, and demographic dimensions. Even though implementation, especially on a national level, might need time, following recent recommendations to collect survey data about e.g. current gender identity in addition to sex assigned at birth [45, 46], would allow health researcher to analyse sex/gender dimensions beyond the standardized binary classification. A more differentiated understanding of sex/gender and its intervowenness with other intersectional variables might have the potential to enhance equity in population health.

The defined criteria for evaluating the included studies in this scoping review might as well be used as a groundwork for conducting and reporting intersectionality-based and sex/gender sensitive quantitative analyses: Embedding research questions into an intersectionality-informed framework might be enhanced by linking the theoretical background to more profound intersectionality related theories (e.g. minority stress), by deciding and describing which and why certain intersectional variables are considered important for analysis and by clarifying why the selected statistical strategy is most suitable for conducting intersectionality based data analysis. With view on sex/gender sensitivity it might be conducive to allocate the research question to one of the central theoretical sex/gender concepts and continuously integrate the perspective in various steps of the research process, e.g. to test hypotheses and to interpret empirical findings. Reflecting on how data about sex/gender was retrieved and what dimensions of sex/gender are actually operationalised as well as including solution-linked variables into multivariable analysis could eventually facilitate the further development and implementation of a gender mainstreaming strategy.

We relied on the term intersect* used in title or abstract of the publications to identify relevant studies. This might be conceived as one limitation of our review, since researcher could be using intersectionality approaches without explicitly mentioning intersectionality as a framework or using respective terminology. The reason most of the included papers originated from the U.S. might as well reflect on how academics in the U.S. are more likely to use the term compared to scholars in other countries doing similar work without explicitly referring to the intersectionality framework. However, we are not aware of any other explicit term which could be used to detect intersectionality-informed studies. Another concomitant limitation of our review is the low number of studies that fulfilled the inclusion criteria. Therefore comparisons across the three thematic fields were not possible. This might be due to the fact that the theoretical intersectionality framework has only recently been introduced into quantitative health research. One strength of our scoping review is the consideration of three distinct health-related thematic fields, diabetes, smoking and physical activity which all play an important role in epidemiology and public health. Finally, the in-depth analysis with regard to theories, operationalisation and statistical methods can be seen as another strength of our review.

## Conclusion

Quantitative studies with an intersectionality-based approach in the thematic fields of diabetes, smoking and physical activity focus mainly on the intersection between sex/gender and race/ethnicity by using them jointly as subgrouping variables and as parts of interaction terms in regression analyses. Despite the fact that sex/gender was the only intersectional-variable considered in every study, it was exclusively operationalised as a binary. Solution-linked sex/gender variables were rarely considered. The theoretical sex/gender concept of Gender was by far the most frequent gender theory referred to, even though the perspective of Gender Equality and Gender Equity might be more strongly linked to the development and implementation of interventions and policies to reduce health inequalities.

Promoting sex/gender-sensitive and intersectional quantitative health research and health reporting from a non-simultaneous intersectional perspective, with sex/gender as the main category of theory and analysis, health research will need to go beyond a mainly explorative approach and to systematically integrate solution-linked variables indicating modifiable aspects of sex/gender-related living conditions and disadvantages. Furthermore, intersectionality-based sex/gender sensitivity in quantitative data analysis might be advanced by consequently considering multiple sex/gender dimensions and their interrelationship with other intersectional-variables. In this regard we would like to suggest to also further theoretical development of Gender, Embodiment, Gender Equality, Gender Equity and Sex as central theoretical sex/gender concepts in health research with intersectionality as an intrinsic perspective, to better theorise and explain between- or within-group differences based on the entanglement of sex/gender with other intersectional-variables.

## Supplementary information

**Additional file 1.** Assignment of explanations for sex/gender differences to respective central theoretical sex/gender concepts (diabetes, smoking and physical activity) (title, description)

**Additional file 2.** Flow chart of all identified studies on title/abstract level (title, description)

**Additional file 3.** Bibliography of included studies (title); List of references of included studies.

**Additional file 4.** Characteristics of selected papers (diabetes, smoking and physical activity) (title); This file shows the characteristics of the included studies in the field “diabetes”, “smoking” and “physical activity”.

## Data Availability

All data generated or analyzed during this study are included in this published article and its supplementary information files.
