# Supplementary material for: Intersectionality-based quantitative health research and sex/gender sensitivity: a scoping review
Source: Int J Equity Health. 2019 Dec 21;18:199. doi: 10.1186/s12939-019-1098-8 (PMC6925460; doi:10.1186/s12939-019-1098-8)
Supplement: Supplementary file 1 — Additional file 1. Assignment of explanations for sex/gender differences to respective central theoretical sex/gender concepts (diabetes, smoking and physical activity) (title, description) [file 12939_2019_1098_MOESM1_ESM.docx]

**Assignment of explanations for sex/gender differences to respective central theoretical sex/gender concepts (diabetes)**

| **Author (year)** | **Sex/gender central theoretical concepts** |
| --- | --- |
| **Assari**  **et al. (2017)** | “Health appraisal involves a series of complex processes of adaptation and personal identity [15]. Evaluation of owns’ SRH is strongly influenced by factors such as race, gender, culture, and class [16]. For Black men, health appraisal may be especially based upon functional status and overall feeling rather than physiologic indicators of health status such as HbA1c or blood pressure [16]”  GENDER INTRA: HEALTH APPRAISEL (INTERSECT: S/G+R/E)  “Health is appraised by comparisons with other people in one’s social network and age group [15, 17, 18]. Because social networks vary in size and quality across race and gender groups, these differences may also shape the interpretation of health in the context of chronic disease [19, 20].”  GENDER INTRA: SOCIAL COMPARISON  GENDER INTER: SOCIAL NETWORKS (INTERSECT: S/G+R/E)  “Finally, there is considerable variation across race and gender in cultural discourses (i.e., effects of culture on communication styles) and individual experiences, which in turn shape individuals’ interpretation of their own health status [21–24].”  GENDER INTER: CULTURAL DISCOURSES (INTERSECT: S/G+R/E)  GENDER INTRA: INDIVIDUAL EXPERIENCES (INTERSECT: S/G+R/E)  “Gender also plays a role, with some data suggesting that women underestimate their health status relative to objective indicators [30, 31]. Additionally, according to the Sponge Hypothesis, women’s SRH is more likely to be influenced by their affect than men’s [30, 31].”  GENDER INTRA: AFFECT  “Gender also significantly affects how individuals evaluate their overall health [16, 30, 32, 37–39]. Compared to men, women are more likely to underestimate their health status, resulting in a weaker overlap between SRH and objective outcomes [30]. The Sponge Hypothesis attributes this variation to the stronger influence of affect on health appraisals of women [38].”  GENDER INTRA: AFFECT |
| **Chesla**  **et al. (2014)** | “Diabetes prevalence is growing fastest in older minority women; women with type 2 diabetes (T2DM) have double the risk of developing coronary artery disease, and have significantly elevated risks of dyslipidemia and hypertension (Legato et al., 2006) compared with men with the disease. Although multiple factors may contribute to gender differences in disease outcomes, perhaps the least attention has been paid to the influence of family and social contextual factors.”  GENDER INTER: FAMILY CONTEXT  GENDER INTER: SOCIAL CONTEXT  “Past research suggests that women respond differently to T2DM than men in three areas: patterns of seeking and receiving support, psychosocial responses to the disease, and gender-related family role expectations. Women are more likely to rely on socially connected forms of information, such as group classes or support groups, whereas men use resources that are not socially mediated, such as the Internet (Mathew, Gucciardi, De Melo, & Barata, 2012). Men report that they rely most heavily on their wives for sup-port, while women rely on female family members and friends. Women with T2DM report less support for their diabetes self-management than men (Cherrington, Ayala, Sacarinci, & Corbie-Smith, 2011; Chlebowy, Hood, & Lajoie, 2013; Mathew et al., 2012), but some studies find no differences (Unden et al., 2008) or greater support reported by women (Misra & Lager, 2009).”  GENDER INTER: SOCIAL SUPPORT  GENDER INTRA: PSYCHOSOCIAL RESPONSES  GENDER INTER: FAMILY ROLE EXPECTATIONS  “Family role expectations, particularly women’s historic role in provision of meals and care of partners and children, negatively influence diabetes management. A meta-synthesis of qualitative studies on diabetes self-management suggests that women take responsibility for diabetes management, but subjugate their needs to the needs of others (Gomersall, Madill, & Summers, 2011). The largest dilemma for women regards the expectation to provide family meals, but not alter the meals to be diabetes-appropriate (Cherrington et al., 2011). Responsibilities to care for children and other family members also impinge on time for diabetes self-management (Gomersall et al., 2011).”  GENDER INTER: FAMILY ROLE EXPECTATIONS  GENDER INTER: PROVISION OF MEALS  GENDER INTER: CARE RESPONSIBILITY  “Here we explore the ways that gender oppression, defined as social and cultural norms, practices and laws that disadvantage women (Ritzer, 2007), is experienced by Chinese American women with T2DM. These women are marginalized by their ethnicity, language, immigrant status, and socioeconomic status (Hankivsky, 2012) and thus gender oppressions introduced by Chinese cultural norms and roles were assumed to intersect with other status and positional inequities (Viruell-Fuentes, Miranda, & Abdulrahim, 2012) to complicate their diabetes experiences.”  GENDER EQUALITY (INTERSECT: S/G+R/E)  Anticipating gender differences in some of these factors in Chinese Americans with T2DM is reasonable given the hierarchical nature of the culture and the privilege accorded men, but this has never been studied.  GENDER EQUALITY (INTERSECT: S/G+R/E)  “Chinese women must navigate intersecting gender and family norms that are shaped by a collectivistic social orientation and Confucian philosophical tenets on family life that prevail in Chinese culture (Sue & Sue, 2003). These norms complicate Chinese women’s ability to address their individual needs and concerns in managing their diabetes (Kwan, Chun, & Chesla, 2011). In Chinese American families, women have less stature in the couple hierarchy, greater responsibility for family emotional well-being, and may feel less emotionally supported in their illness. Normatively, Chinese women are more attuned to, concerned about, and responsible for maintaining family harmony; to maintain harmony, they avoid family conflict and thus may experience greater unresolved family conflict than do their male counterparts (Kawahara & Fu, 2007).”  GENDER INTER: FAMILY ROLE EXPECTATIONS (INTERSECT: S/G+R/E)  “Adherence to family role responsibilities are highly valued in Chinese culture (Kim, Yang, Atkinson, Wolfe, & Hong, 2001; Lee, Choe, Kim, & Ngo, 2000) and individuals may experience shame and loss of face if they fail to meet family expectations. Chinese women’s role expectations include primary responsibility for childrearing and maintaining the home (Chin, 2000), although work outside the home is also common in immigrants. Thus, gender and family role expectations may disadvantage Chinese immigrant women regarding the time, attention, and support they receive in meeting personal diabetes needs and concerns.”  GENDER INTER: GENDER ROLE EXPECTATIONS (INTERSECT: S/G+R/E)  GENDER INTER: FAMILY ROLE EXPECTATIONS (INTERSECT: S/G+R/E)  “Attributing women’s elevated distress to gender oppression alone is, of course, insufficient. Distress arises from women’s holistic experience of accommodating diabetes as immigrants, with relatively few resources, and often in a context of language discordance.”  GENDER EQUALITY (INTERSECT: S/G+R/E)  “However, the observation that women who were in similar social and disease situations as their male counterparts appeared to suffer greater psychological distress suggests that first-generation Chinese American women are affected by gender oppression.”  GENDER EQUALITY (INTERSECT: S/G+R/E)  “Women reportedly prefer socially connected approaches to improving their diabetes knowledge and skill (Mathew et al., 2012), although this has never been established in Chinese Americans.”  GENDER INTER: SOCIAL SUPPORT  “Given Chinese women’s traditionally less-powerful position in the family, it is likely that focusing on their own struggles empowered women to recognize personal needs, and to negotiate ways to meet them.”  GENDER EQUALITY (INTERSECT: S/G+R/E)  “Appreciating the multiple contextual factors that may contribute to this distress, rather than focusing only on individual psychological difficulties, enables a more empathic and multifaceted approach to women’s health care. Second, group behavioral interventions directed toward not only illness management but also toward strengthening skills for handling family and social dilemmas may particularly resonate for Chinese American women, although positive effects were noted in both genders”  GENDER INTER: DISTRESS DUE TO CONTEXTUAL FACTORS (INTERSECT: S/G+R/E)  GENDER EQUITY |
| **Gagne & Veenstra (2017)** | “Our results suggest that poor Black women in Canada experience a multiplicative jeopardy of hypertension that is higher than expected given the distinct jeopardies of being poor, being Black, and being women. These results are troubling in light of the economic inequalities that continue to negatively affect Black Canadians. Similar to American trends, it could be that poor Black women are more likely than poor White Canadians and poor Black men to be single parents, have long commutes to work, work at menial jobs, or lack access to quality health care. For instance, in the province of Nova Scotia, many Black Canadians live in rural settings with a limited health service infra-structure providing access to preventive measures that mitigate the development of health conditions such as hypertension.30-32 Enang notes that a high proportion of Black women in that province are not able to avail themselves of preventive health tests such as Pap smears, breast exams, and routine physical examinations; this may be especially true for poor Black women.33 These women may also encounter expectations to be strong role models for their families and others in the face of everyday racism and/or may have fewer resources to maintain resiliency in the face of racism.”  GENDER INTER: ECONOMIC INEQUALITIES  GENDER INTER: EXPECTATIONS TO BE STRONG ROLE MODELS FOR THEIR FAMILIES (INTERSECT: G+R/E+I)  GENDER INTRA: RESILIENCY (INTERSECT: S/G+R/E+I)  “In regard to the absence of relationships between income and both health outcomes for Black Canadian men, similar results have been noted in the United States with regard to diabetes and hypertension among African Americans and Black Americans of Caribbean origin.6,34-37 Robbins and colleagues examined 1,800 Black American participants in the US National Health and Nutrition Examination Survey (NHANES III) and found no significant association between income and diabetes among African American men and a strong association between income and diabetes among African American women. They suggested that different experiences of obesity, stress, and depression could contribute to explaining these gender differences. In Canada, evidence suggests that Black women and men face a similar burden of discrimination in everyday life and the workplace.37 However, it might be the case that wealthy Black men work in more predominately White environments, which puts them at higher risk of experiencing unique kinds of health-damaging discrimination.”  GENDER INTER: DISCRIMINATION (INTERSECT: S/G+R/E) |
| **Salsberry**  **et al. (2007)** | “Another interpretation of these findings is that there is a protective effect of higher income for women but not men. This is also noteworthy inasmuch as men with high incomes experience each of the individual risks at about the same rate as men in the lowest economic group. It may also be that social pressures on women with high incomes are different from those on high- income men and that obesity is less acceptable in high-income women compared with men.34,35 Women in high-income households are also less likely to participate in the labor market than either women in lower-income households or men, which may reduce overall levels of stress among this particular group.36”  GENDER INTER: SOCIAL PRESSURE ON HIGH-INCOME WOMEN VS HIGH-INCOME MEN (INTERSECT: S/G+I)  GENDER INTER: PARTICIPATION IN LABOUR MARKET (INTERSECT: S/G+I)  GENDER INTER: STRESS (INTERSECT: S/G+I)  “Black women in this study were no more likely to have metabolic syndrome than white women, and they were substantially less likely to be at risk for elevated triglycerides. Black men were the least likely to experience metabolic syndrome. This was driven by the lowest rates of abdominal obesity and low rates of elevated glucose and triglycerides. These findings of reduced risk for elevated triglycerides in blacks confirm results from other studies.38–41 Changes in lipid metabolism might also explain the lower level of central obesity in black males found in the current study. Although the genetic profile of black women is also favorable in regard to lipid expression, this benefit is obscured by greater central obesity and higher risk for hypertension compared with white women.”  SEX: GENETIC PROFILE (INTERSECT: S/G+R/E) |
| **Walmer**  **et al. (2015)** | “Anxiety and depressive disorders are highly prevalent in the general population and women of childbearing age are particularly susceptible from delivery through 1-year postpartum.[11]”  SEX: CHILDBEARING  “For example, although we observed significantly lower rates of depression, anxiety, and other mental health disorders in the postpartum period for Asian women compared to white women, our study was not designed to determine if these findings were attributed to cultural stigma limiting individual reporting; a dearth of bilingual providers to capture this medical history; or the participants’ mistrust of mental health service providers limiting disclosure of this information.”  GENDER EQUALITY: CULTURAL STIGMA (INTERSECT: S/G+R/E)  GENDER EQUITY: BILINGUAL PROVIDER TO CAPTURE MEDICAL HISTORY (INTERSECT: S/G+R/E) |

**Assignment of explanations for sex/gender differences to respective central theoretical sex/gender concepts (smoking)**

| **Author (year)** | **Sex/gender central theoretical concepts** |
| --- | --- |
| **Aguirre**  **et al. (2016)** | “Rather, the combination of being both a woman and a member of an ethnic minority group may generate a qualitatively unique psychosocial context that increases the likelihood of a specific pattern of health behavior that is not adequately reflected by exploring the independent/ additive effects of gender and ethnicity.”  GENDER INTER: PSYCHOSOCIAL CONTEXT (INTERSECT: S/G+R/E)  GENDER INTRA: HEALTH BEHAVIOUR? (INTERSECT: S/G+R/E)  EMBODIMENT (SOCIAL) (INTERSECT: S/G+R/E)  “Because of possible gender-specific ethno-cultural variation, such intersectionality in smoking expectancies is plausible. For instance, the salience of smoking’s expected effects on weight control may be more salient for non-Hispanic White (vs. non-Hispanic African American) women because culturally specific ideals that value thinness among women may be strong in non-Hispanic Whites, yet weaker in non-Hispanic African American populations in which cultural norms for women’s body shapes place less value on thinness (Kronenfeld, Reba-Harrelson, Von Holle, Reyes, & Bulik, 2010; Molloy & Herzberger, 1998; Rogers Wood & Petrie, 2010; Watson, Ancis, White, & Nazari, 2013).”  GENDER INTER: CULTURAL NORMS FOR WOMEN’S BODIES (INTERSECT: S/G+R/E)  “Moreover, identifying gender-by-ethnicity interactions in the prediction of smoking expectancies may facilitate in the understanding of underlying cognitive mechanisms that may drive tobacco-related health disparities.”  GENDER INTRA: COGNITIVE MECHANISMS (INTERSECT: S/G+R/E)  “We also found evidence of possible intersectionality between gender and ethnicity on weight control (but not other forms of) smoking expectancies. Weight control smoking expectancies were stronger in women versus men in non-Hispanic Whites, but did not differ by gender among Hispanics or non-Hispanic African Americans. A prior study in adolescents showed that non-Hispanic White adolescent girls reported smoking to lose or control weight as a reason for smoking more often than Hispanic and non-Hispanic African American girls (Fulkerson & French, 2003). Cultural differences in acceptance of a wider range of body shapes and sizes for women may influence the salience of smoking’s weight controlling effects as a function of ethnicity.”  GENDER INTER: CULTURAL NORMS FOR WOMENS’S BODIES |
| **Bilal**  **et al. (2016)** | “Recently, female empowerment has been shown to be correlated with an increased prevalence of smoking among women relative to men6 and may be one of the factors that—together with industry activity7—shape the population distribution of smoking.”  GENDER EQUALITY  “First, we approach the evaluation of smoking prevalence differences from a gender perspective that treats these differences as the result of social processes operating at an intrapersonal, interpersonal and society level.8 These processes relate to both socioeconomic and gender differentials, which are known to be strong determinants of smoking prevalence.9 Second, we acknowledge that smoking may be one of the pathways that embody unequal power structures.”  GENDER INTER: SOCIAL PROCESSES (INTERSECT: S/G+I)  GENDER EQUALITY  EMBODIMENT (SOCIAL)  “The observed cohort effect in both male and female smoking prevalence patterns suggests that women born in a more gender equal context have smoking prevalence patterns that emulate those of men.”  GENDER EQUALITY  “The association between gender inequality and GSR can be explained by the rapid social, political and economic transformation that took place in Spain over the past 50–60 years, including a rapid evolution and improvement in the living conditions and opportunities of women living in Spain.14 These changes created opportunities for the tobacco industry to specifically target women using emancipation imagery, depicting smoking as a symbol of success and gender equality.7 Previous analyses of internal documents and advertisements in Spain19 have shown that tobacco companies started targeted advertising towards women in the early-to-mid 1980s, an era of enormous social change in Spain that included an increase in women’s participation in the labour market and the loosening of social constraints on smoking.14”  GENDER EQUALITY  GENDER INTER: PARTICIPATION IN LABOUR MARKET  “Gender-sensitive policies13 are needed in countries in earlier stages of the epidemic in order to keep smoking prevalence low in both men and women. Moreover, special focus should be given to low SES women.”  GENDER EQUITY  GENDER EQUITY (INTERSECT: S/G+I)  “Our findings indicate that an increase in gender equity in Spain throughout the five decades studied (1960–2010) was followed by an increase in the female-to-male smoking ratio, with differential effects by birth cohort and education level. Given the health effects of smoking, gender-sensitive tobacco control measures, policies and programmes are necessary to protect women, especially those of low SES, from the burden of smoking.”  GENDER EQUITY  GENDER EQUITY (INTERSECT: S/G+E) |
| **Cubbin**  **et al. (2010)** | - |
| **Gaalema**  **et al. (2018)** | - |
| **Klassen**  **et al. (2015)** | “Although ethnic disparities are often speculated to be driven at least in part by social and economic resource differentials [15], there is incomplete understanding of the race-specific role of social resources on breast cancer outcomes, and whether specific behavioral correlates of social class actually drive biological outcomes.”  GENDER INTRA: BEHAVIOUR (INTERSECT: S/G+R/E+I)  EMBODIMENT (SOCIAL) (INTERSECT: S/G+R/E+I)  “Breast cancer disparities, at their most essential, represent the biological manifestation of a myriad of physical, environmental, social and behavioral differences between women.”  GENDER INTER: PHYSICAL DIFFERENCES  GENDER INTER: ENVIRONMENTAL DIFFERENCES  GENDER INTER: SOCIAL DIFFERENCES  GENDER INTRA: BEHAVIORAL DIFFERENCES  EMBODIMENT (ECO-SOCIAL)  “However, our analyses suggest that social class resources are not equally protective for black and white women, in that black women received less protection from higher social class in regard to later stage at diagnosis, more aggressive histological grade, and larger tumor size. This is consistent with theories of social stratification which focus on intersectionality, or the combined effects of race, gender, and social resources. Intersectionality theory [40] would argue that the meaning of a certain social achievement, for example college graduation or white collar employment, cannot be determined out of context from other social factors, and will confer different social advantage for women than men, and minority compared to majority racial groups. In the case of these analyses, a myriad of behavioral, environmental, or early detection-related factors may differ more between socially advantaged and disadvantaged white communities than those in which black cases reside.”  GENDER INTER: SOCIAL CLASS RESOURCES (INTERSECT: S/G+R/E)  GENDER INTER: SOCIAL ADVANTAGE |
| **Ortiz**  **et al. (2018)** | ~~-~~ |
| **Pang**  **et al. (2018)** | “Tobacco withdrawal is an important factor that maintains tobacco addiction and impedes smoking cessation success.11,12 One motivationally prepotent feature of the tobacco withdrawal syndrome is negative affect, which is predictive of increased risk of smoking reinstatement in the lab and smoking maintenance and relapse during cessation attempts.12,13 Given its formative influence on the motivational salience of smoking reinstatement among chronic smokers, it is possible that withdrawal-related negative affect experienced during tobacco abstinence may underlie the relatively higher odds of cessation failure and tobacco-related health problems observed among both women (vs. men) and African Americans (vs. other racial backgrounds).”  GENDER INTRA: AFFECT  “Several laboratory and clinical studies indicate that women compared to men report greater abstinence-induced severity of negative affect states, withdrawal-related distress, and expectations that smoking relieves negative affect.13–16 Moreover, recent epidemiological data suggest that women are more likely than men to endorse a greater variety of tobacco withdrawal symptoms (eg, anxiety, irritability) and may also exhibit greater withdrawal-provoked discomfort and relapse.10Gender differences in withdrawal-related negative affect have been shown to mediate the effects of gender on smoking behavior.13,14Hence, extant work indicates that changes in negative affect states during tobacco abstinence may be particularly important for understanding gender-related health disparities in smoking behaviors and cessation success. Yet, it remains unknown whether these gender differences in tobacco withdrawal generalize across different racial groups.”  SEX: GREATER VARIETY OF TOBACCO WITHDRAWAL SYMPTOMS  GENDER INTRA: AFFECT  “Research examining whether gender differences in affect following acute tobacco abstinence distinctly manifest or generalize across racial groups remains scarce. However, a “sociopharmacological” model of tobacco-related health disparities20 proposes that psycho-biological and sociocultural factors that differ by race or gender (eg, nicotine metabolism, expression of affect) may moderate both the overall averseness of the withdrawal experience and the extent to which tobacco’s affect-modulating effects translate into conscious motivation to relieve affective distress via reinstating smoking.”  EMBODIMENT (PSYCHO-BIOLOGICAL) (INTERSECT: S/G+R/E)  GENDER INTER: SOCIOCULTURAL FACTORS (INTERSECT: S/G+R/E)  SEX: NICOTINE METABOLISM  GENDER INTRA: AFFECT (INTERSECT: S/G+R/E)  “There are a number of possible biological and sociocultural mechanisms that might account for elevated sensitivity to tobacco abstinence–related negative affect among non-Hispanic White women smokers. Prior research has found that hormonal factors in women (eg, menstrual cycle, ovarian hormones, hormonal medication) impact negative affect during acute tobacco abstinence31–34 and that hormone dynamics may differ between non-Hispanic African American and non-Hispanic White women.35 One limitation of this study is its lack of direct hormonal assessment, and thus, we are unable to determine the potential influence of hormones in this study. It will be important for future studies to address the potential role of racial differences in hormonal factors on tobacco abstinence–related negative affect withdrawal. Sociocultural factors may also potentially underlie racial differences in the impact of gender on tobacco abstinence–related negative affect. Factors related to cultural socialization may render African Americans (vs. White Americans) less likely to present emotional disturbance in terms of negative affect.36 Moreover, a prior study found gender differences in emotional expressions among Whites, but not non-Hispanic African Americans.37 Thus, it is possible that differences in cultural norms surrounding affective expression may account for the race–gender patterns of tobacco abstinence–related negative affect observed in our sample.”  SEX: HORMONAL FACTORS (INTERSECT: S/G+R/E)  GENDER INTRA: BIOLOGICAL MECHANISMS (INTERSECT: S/G+R/E)  GENDER INTER: SOCIOCULTURAL MECHANISMS/FACTORS (INTERSECT: S/G+R/E)  GENDER INTER: CULTURAL NORMS (INTERSECT: S/G+R/E)  GENDER INTRA: EMOTIONAL EXPRESSIONS (INTERSECT: S/G+R/E)  “In summary, non-Hispanic White women experience increased exacerbations of negative affect following acute tobacco abstinence in comparison to non-Hispanic White men, but these gender differences were not found in non-Hispanic African American smokers. Given recent findings suggesting that slower dissipation of negative affect (particularly anxiety and anger) may impact smoking cessation outcomes,44 these results are significant because they provide further evidence that non-Hispanic White women (vs. non-Hispanic White men) smokers may be susceptible to smoking cessation failure due to increased negative affect. Although we found smaller-sized abstinence-induced changes in negative affect in our study and are currently unaware of an exact cutoff for changes that would be of clinical significance, prior cessation studies have demonstrated that small changes in affect predict early versus late lapsers during cessation;45,46 despite smoking thus, one clinical implication of this finding is that small to modest changes in negative affect could precipitate earlier relapse during cessation among non-Hispanic White women. These studies also suggest that prior findings demonstrating the central role of negative affective withdrawal as a driving factor of smoking maintenance in women compared to men may not generalize to non-Hispanic African American smokers. However, several studies in samples of non-Hispanic African American smokers have found that women report expectations of smoking-induced negative affect relief and stress reduction.29,47,48 Thus, even though non-Hispanic African American women did not report greater abstinence-induced negative affect in this study, it is possible that negative reinforcement- mediated smoking may still be an important mechanism underlying tobacco addiction in African American women, and it is essential that future work continue to explore etiological mechanisms underlying tobacco addiction among non-Hispanic African American women smokers. In addition, future research should address whether the racially discrepant patterns of gender differences in negative affect–related tobacco withdrawal reported in this study are found during self-motivated quit attempts. Such work may inform empirical efforts to develop tailored, gender- and race-specific cessation strategies to improve smoking outcomes in vulnerable groups subject to tobacco-related disparities.”  GENDER INTRA: AFFECT (INTERSECT: S/G+R/E) |
| **Villanti**  **et al. (2018)** | - |

**Assignment of explanations for sex/gender differences to respective central theoretical sex/gender concepts (physical activity)**

| **Author (year)** | **Sex/gender central theoretical concepts** |
| --- | --- |
| **Abichahine &**  **Veenstra (2017)** | “In short, evidence from Canada appears to assign regular physical activity to well-educated and wealthy White men above all others and to poorly educated and poor visible minority women least of all. Presumably the social privilege inherent to being of high socioeconomic status, White or male is accompanied by factors such as access to and availability of leisure time, autonomy and flexibility in the workplace and access to childcare that facilitate engaging in leisure-based physical activity in everyday life.”  GENDER INTER: SOCIAL PRIVILEGE  GENDER INTER: WORKPLACE CONDITIONS  GENDER INTER: ACCESS TO CHILDCARE |
| **Ray**  **(2017)** | “If these spaces are racialized or gendered, physical activity may be less prevalent for certain groups embedded in certain social contexts, while physical activity may be more prevalent for certain groups embedded in different social contexts.”  GENDER INTER: SOCIAL CONTEXT (INTERSECT: S/G + R/E)  “Similar to black women, black men's identities straddle the intersection of race and gender. While black women experience sexualization, black men are more prone to experience criminalization.”  GENDER INTER: SEXUALISATION (INTERSECT: S/G+R/E)  GENDER INTER: CRIMINALIZATION (INTERSECT: S/G+R/E)  “Although middle class white men as well as women (as women are primarily affected by safety and perceptions of safety more than men) may engage in more physical activity in predominately white neighborhoods (Bennett et al., 2007), black men may experience a heightened level of criminalization in predominately white neighborhoods that results in less physical activity. Research on black men in public spaces asserts that predominately white environments lead to a heightened level of visibility and racial profiling for black men that may result in less community engagement (Feagin and Sikes, 1995; Houts Picca and Feagin, 2007; Feagin, 2010; Ray and Rosow, 2012). In his examination of the social relations between whites and blacks in the 21st century, Feagin (2010) finds that whites have limited social class cues to tell differences among black men (i.e., professor, lawyer, delivery man, criminal). Feagin states, “Many whites have fearful reactions to a black man encountered on streets, in public transport, and in elevators” (Feagin, 2010, p.108). Compared to other race-gender groups, McConnaughy and White (2011) find that whites perceive black men as more violent, unpleasant, promiscuous, unintelligent, and less ambitious and nurturing. Psychologists document that some whites are more likely to perceive black men as aggressive, have a similar fear of black men as they do of snakes and spiders, and are more likely to pull the trigger of a gun quicker on an unarmed, black man compared to an unarmed, white man, and even at times an armed white man (Eberhardt et al., 2004; Correll et al., 2006; Trawalter et al., 2008). Consequently, most black men become criminalized, even middle class black men who live next door to whites in middle class neighborhoods.”  GENDER INTRA: PERCEPTIONS OF NEIGHBORHOOD SAFETY  GENDER INTER: CRIMINALIZATION (INTERSECT: S/G+R/E)  GENDER INTER: STIGMATIZATION  GENDER INTRA: COMMUNITY ENGAGEMENT (INTERSECT: S/G+R/E)  “Middle class black men's experiences with whites start long before they move into predominately white neighborhoods. As teenagers, blacks are more likely to be perceived as adults (Rattan et al., 2012). Experiences in college often help shape middle class black men's perceptions of interacting in predominately white environments. While collegiate white men are able to gain purchase on their high-status because a white racial identity affords them certain advantages in public spaces and predominately white environments (e.g., ability to blend into a crowd, interact as individuals, and experience a lack of accountability for the behavior of other white men), collegiate black men experience a hyper-level of visibility and more policing in these same spaces (Ray and Rosow, 2012; Ray, 2013). This heightened level of visibility increases the likelihood of unjust treatment, which leads to more stress and less community engagement. If black men are less likely to engage in their community due to profiling and policing, they also may be less likely to walk, run, or engage in other forms of leisure-time physical active in these neighborhoods.”  GENDER INTER: STIGMATISATION (INTERSECT: S/G+R/E)  GENDER INTER: CRIMINALIZATION (INTERSECT: S/G+R/E)  GENDER INTRA: STRESS DUE TO UNJUST TREATMENT (INTERSECT: S/G+R/E)  GENDER INTRA: COMMUNITY ENGAGEMENT (INTERSECT: S/G+R/E)  “Although middle class black men may be less physically active in predominately white neighborhoods, they may actually be more physically active in predominately black neighborhoods. Predominately black neighborhoods may provide a level of comfort as black men are less likely to be hyper-visible and racially marked (Pattillo-McCoy, 1999). Some of these public spaces include parks and gyms. While predominately black neighborhoods are often less safe, Bennett et al. (2007) find that safety concerns do not affect men's level of physical activity.”  GENDER INTER: STIGMATIZATION (INTERSECT: S/G+R/E)  GENDER INTRA: SAFETY CONCERNS  “Nonetheless, the benefits that predominately black neighborhoods afford black men in terms of comfort for leisure-time physical activity may not exist for black women. First, women, unlike their male counterparts, are affected by safety concerns. Bennett et al. (2007) find that women are significantly less likely to be physically active as safety concerns increase. Second, well-resourced neighborhoods, which are also more likely to be middle class and predominately white, have facilities and programs that cater specifically to women. These “women-only” zones may provide protection from “catcalling” and the male gaze while women engage in physical activity (Ray, 2014).”  GENDER INTRA: SAFETY CONCERNS  GENDER EQUITY (INTERSECT: S/G+SES)  “Third, middle class black women receive fewer returns on their education for income and are more likely to be single mothers than other race-gender groups in the middle class (Cummings and Jackson, 2008; Ray, 2014). Consequently, they may have less income to devote to the housing market. In turn, their neighborhoods may have fewer resources and less safety than other individuals in the middle class. On average, predominately black neighborhoods, regardless of social class composition, have fewer facilities and programs that cater to the childcare needs of working mothers (Charles, 2003). Childcare is important considering that the family-work life literature shows “that women are still pulling the ‘second shift’ at home by having to do most of the caregiving and housework after they come home from their paid jobs. Black women, compared to white women, are more likely to work full-time, less likely to be married, and more likely to have children in the home” (Ray, 2014, p. 783).”  GENDER EQUALITY (INTERSECT: S/G+R/E)  GENDER EQUITY (INTERSECT: S/G+R/E)  GENDER INTER: CARE RESPONSIBILITIES  GENDER INTER: HOUSEWORK RESPONSIBILITIES  “For women, however, safety as a barrier to physical activity may not only capture perceptions of being victims of crime but also gendered and sexualized vulnerabilities of trying to engage in physical activity in dense spaces where they may experience catcalling and the male gaze (Ray, 2014); or as bell hooks (1992) states, the ability for men to rape women with their eyes. This is not to say that this type of behavior and interaction between men and women does not occur in predominately white spaces. Evidence exists on the vulnerabilities of women in male-dominated spaces on predominately white campuses (Ray and Rosow, 2010; Armstrong and Hamilton, 2013). For physical activity, however, more well-resourced neighborhoods, which according to my findings are more likely to be perceived as predominately white, are more likely to have spaces where women can engage in physical activity without the purview of men. For example, fitness centers in more affluent neighborhoods are creating “women-only zones” where women can forgo male-dominated spaces (Ray, 2014). Some facilities, such as Curves Fitness, cater specifically to women. These types of businesses, however, may be less present in neighborhoods perceived as predominately black.”  GENDER INTRA: SAFETY CONCERNS  GENDER INTER: SEXUALIZATION  GENDER EQUITY (INTERSECT: S/G+R/E)  “Black men have a different social reality from their black female counterparts. Supporting literature on criminalization (Feagin and Sikes, 1995; Houts Picca and Feagin, 2007; Feagin, 2010; Ray and Rosow, 2012), black men are significantly less likely to be physically active in neighborhoods perceived as predominately white. Although middle class black men may experience policing in predominately black neighborhoods, they may not feel or experience the safety and comfort of well- resourced, predominately white neighborhoods either. Experiencing discrimination or relative deprivation at a neighborhood business such as a restaurant or store may have a spillover effect to other spaces like gyms or parks, which may in turn, decrease physical activity and community engagement.”  GENDER INTER: CRIMINALIZATION (INTERSECT: S/G+R/E)  GENDER INTER: DISCRIMINATION (INTERSECT: S/G+R/E)  GENDER INTER: RELATIVE NEIGHBORHOOD DEPRIVATION  “As a coping response to experiencing criminalization, middle class black men may undergo a signaling process where they feel the need to signal their middle class status in predominately white spaces. For example, Staples (1986) discussed how he would whistle melodies from classical composers while walking through Hyde Park to the University of Chicago in order to make whites feel less threatened by his presence. For middle class black men engaging in leisure-time physical activity, this signaling process may mean always carrying a driver's license or other form of identification, wearing an alumnus shirt of a notable university, walking along busy or well-lit streets, running during daylight hours, and smiling and waving at neighbors. Consequently, this signaling process may lead to less leisure-time physical activity for middle class black men in neighborhoods perceived to be predominately white.”  GENDER INTRA: COPING RESPONSE TO CRIMINALIZATION (INTERSECT: S/G+R/E)  “Unlike black men, whites may not undertake (or feel the need to undertake) this signaling process when they simply want to run or walk a couple of miles around their neighborhood. Although facing their own stereotypes, black women are not criminalized as being violent predators to the same degree as black men (McConnaughy and White, 2011). In turn, they are less threatening to the public, social order of predominately white neighborhoods.”  GENDER INTER: CRIMINALIZATION (INTERSECT: S/G+R/E)  “The signaling process that middle class black men undergo has implications for self-presentation theory (Goffman, 1959). Middle class black men in neighborhoods perceived to be predominately white are aiming to present themselves as having earned the right to belong like their white male counterparts. Goffman (1959) states that the initial definition of the situation is determined by preconceived notions or inferences about the other in the social interaction. Middle class black men are well aware of the negative stereotypes about black men (Feagin and Sikes, 1995; Ray and Rosow, 2012). By aligning their self- presentations with the norms of predominately white neighborhoods, middle class black men are aiming to counter the negative inferences about their race-gender group (Staples, 1986; Ray and Rosow, 2012). Unfortunately, research shows that black men's social class cues are unable to substitute for the intersection of their black skin and maleness. As part of their self- presentation, middle class black men's intersectional identity is unable to overcome the main inference that provides relief from negative stereotyping and this signaling process - an ideal white racial identity (Hughey, 2012).”  GENDER INTER: STEREOTYPING (INTERSECT: S/G+R/E)  “Conversely, neighborhoods perceived to be predominately black (even those perceived as having less safety and fewer facilities and programs focused on leisure-time physical activity) may allow black men to engage in the community without experiencing a heightened level of visibility and racial stereotyping. Given the high level of racial segregation in the United States, a majority of black males grow up in and around predominately black neighborhoods (Pattillo-McCoy, 1999). Unlike predominately white neighborhoods where a black male face becomes a perceived threat to the social order (Feagin, 2010; Ray, 2015), predominately black neighborhoods provide black men a sense of belonging. In turn, they may increase their community engagement and leisure-time physical activity.”  GENDER INTER: STEREOTYPING (INTERSECT: S/G+R/E)  GENDER INTRA: COMMUNITY ENGAGEMENT (INTERSECT: S/G+R/E)  “My research also has implications for scholars interested in intersectional identities (Cummings and Jackson, 2008; Wilkins, 2012; Bauer, 2014; Ray, 2014; Brown et al., 2016). Perceptions of race, gender, and social class identities guide individuals' interactions with others. Perceptions of the most salient feature(s) of an intersectional identity may be significant at determining social interactions and their consequences. Middle class black men perceive that the intersection of their race and gender identities frequently trumps their social class identity. The perceptions of others constrain black men's social world and influence black men's social interactions with co-workers and neighbors. As a result, these perceptions structure a unique form of relative deprivation that not only leads to less physical activity in neighborhoods perceived to be predominately white but also a different set of coping responses to unjust treatment, benign neglect, and perceived discrimination. In this regard, the intersectionality framework becomes useful for illuminating black men's multiplicities and vulnerabilities.”  GENDER INTRA: SOCIAL INTERACTIONS (INTERSECT: S/G+R/E)  GENDER INTRA: SOCIAL CLASS IDENTITY (INTERSECT: S/G+R/E)  GENDER INTRA: GENDER IDENTITY (INTERSECT: S/G+R/E)  GENDER INTRA: RACE IDENTITY (INTERSECT: S/G+R/E)  GENDER INTER: DISCRIMINATION (INTERSECT: S/G+R/E)  GENDER INTRA: COPING RESPONSES TO UNJUST TREATMENT (INTERSECT: S/G+R/E)  GENDER INTRA: SOCIAL INTERACTIONS (INTERSECT: S/G+R/E) |
| **Wells**  **et al. (2017)** | “Health inequalities may, for example, develop from structural disadvantages imparted on women and ethnic minority or immigrant groups (Graham, 2009). Female gender is associated with adolescent physical inactivity (for a review, see Sallis, Prochaska, & Taylor, 2000) and socioeconomic advantage may positively affect girls’ participation in physical activity more so than boys’, though the latter is not a consistent finding (Hanson & Chen, 2007). Few studies have investigated whether immigrant background may be associated with young people’s physical inactivity. Yet there is indication that immigrant background is associated with adolescent physical inactivity in the United States (Singh, Stella, Siahpush, & Kogan, 2008), Canada (Kukaswadia, Pickett, & Janssen, 2014), and Sweden, though only for girls (Gillander Gådin & Hammarström, 2002; Kahlin, Werner, Romild, & Alricsson, 2009). It is unknown whether this association may persist in young adulthood or whether it could be explained by socio-economic position.”  GENDER EQUALITY  GENDER INTER: SOCIO-ECONOMIC POSITION  “Numerous studies have reported that adolescent girls are at greater risk for physical inactivity than boys (Sallis et al., 2000), and our results corroborate this finding. Adolescent girls were significantly more likely to be inactive in leisure- time organized physical activity, which Vilhjalmsson and Kristjansdottir (2003) have argued is an important dimension of physical inactivity with regard to gender, as the often-cited gender difference in adolescent physical activity may be attributable to a gender divide in sports club enrolment. That girls are less likely to join sports clubs may be due to greater socialization of boys into organized sports and differential encouragement given to boys over girls to value competition and athleticism (Vilhjalmsson & Kristjansdottir, 2003). However, we also found that being male translated into a greater risk of physical inactivity in young adulthood. Gender differences in physical inactivity in this age-group are largely unexplored in the literature; however, our results are in line with a longitudinal Finnish study that found that boys’ physical activity levels decline more quickly than girls from early adolescence to young adulthood (Telama & Yang, 2000). It is also plausible that our more global measure of physical inactivity in young adulthood better captures the physical activities in which women engage (i.e., more independent and less competitive activities like walking; Vilhjalmsson & Kristjansdottir, 2003). Of note, gender differences in physical inactivity at both time points in this study were not dependent on how we dichotomized the physical inactivity variables.”  GENDER INTER: GENDER NORMS  “Further addressing our first two research questions, we found that physical inactivity was more systematically patterned by socioeconomic position, that is, in expected directions, for girls than boys. We found girls to be at greater risk for physical inactivity if their parents held low educational attainment, were not higher nonmanual workers, and had low income. Among these, social class and income were the most important dimensions in adolescence and education was the most important in young adulthood. The patterning among boys did not correspond with socioeconomic disadvantage as systematically. However, boys in families with lower income were more likely to be physically inactive in adolescence and a similar tendency persisted in young adulthood, though it was not statistically significant in the full model. Identifying why specific dimensions of socioeconomic position may impact girls versus boys differently, and during different developmental periods in youth, is a complex endeavour. For instance, it is unclear why only young men with self-employed parents would be at a greater risk of physical inactivity (finding not significant in the full model). It is also difficult to disentangle why adolescent boys with upper secondary educated or intermediate/lower nonmanual parents would be less likely to be inactive compared with boys whose parents have tertiary education or are higher nonmanual workers, respectively (of note, results were similar when checked in the larger sample of adolescents who participated in the Child-LNU). Interpreting results for girls was more straightforward. That parental social class and income largely explained the association between parental education and adolescent girls’ physical inactivity can be partially understood in terms of how different dimensions of socioeconomic position are acquired in the parental generation. As education provides qualifications for more desirable occupations with high salaries, social class and income can be regarded as mediators (or are more important per se) in the association between education and physical inactivity. More specifically, not belonging to the higher nonmanual social class may translate into fewer opportunities for adolescents to participate in organized activities (perhaps due to differential class norms or neighbourhood disadvantage), and it is not unreasonable to expect that if opportunities are few that this would affect girls’ participation more so than boys’, as boys are socialized to participate in sports to a greater degree (Vilhjalmsson & Kristjansdottir, 2003). Additionally, the impact of disposable income on adolescent physical inactivity (found for girls and boys) may be understood in terms of the financial investment often required for enrolment in leisure-time athletic activities. It is less clear why we find a strong independent effect of parental education on young women’s physical inactivity in young adulthood. However, there is evidence that education, when compared with other dimensions of socioeconomic position, is more strongly connected to adult women’s health (Torssander &Erikson, 2010) and health behaviours (Dorner, Strogenner, Hoffmann, Stein, & Niederkrotenthaler, 2013). It is possible that higher education equips parents with a greater awareness of the importance of physical activity and gender equality, which could translate into a greater likelihood for their daughters to participate in physical activities and go on to achieve a high education themselves (which would then in turn also be associated with physical activity). However, more research is needed testing such mechanisms.”  GENDER-INTRA: HEALTH-RELATED BEHAVIOUR (INTERSECT: S/G + SES)  GENDER INTER: GENDER NORMS  GENDER EQUALITY  “We also found that immigrant background was associated with physical inactivity in adolescence (for girls only) and in young adulthood (both genders). A synergistic interaction between immigrant background and female gender in adolescence lent further support to an intersectional interpretation, where being female and having an immigrant background translate into a higher proportion of physically inactive adolescents that is greater than summing the independent effects of being female and having an immigrant background (Bauer, 2014; Kalilani & Atashili, 2006). This finding also supports previous research conducted in Sweden that found an association between immigrant background and physical inactivity for adolescent girls only (Gillander Gådin & Hammarström, 2002; Kahlin et al., 2009). Possible explanations for this association include differences in socioeconomic position (of which we find some support) and other mechanisms outside the scope of this study, including differential norms held by parents regarding girls’ participation in physical activity and discrimination or exclusion of girls with an immigrant background from participating in leisure-time organized athletic activities. However, as we also find that immigrant background is associated with physical inactivity for both men and women in young adulthood, further research should consider how such mechanisms may be specific to time period in youth or type of physical inactivity.”  GENDER: SOCIOECONOMIC POSITION (INTERSECT: S/G + R/E)  GENDER INTER: GENDER NORMS  GENDER INTER: DISCRIMINATION (INTERSECT: S/G + R/E) |
