# Supplementary material for: Intersectionality-based quantitative health research and sex/gender sensitivity: a scoping review
Source: Int J Equity Health. 2019 Dec 21;18:199. doi: 10.1186/s12939-019-1098-8 (PMC6925460; doi:10.1186/s12939-019-1098-8)
Supplement: Supplementary file 2 — Additional file 2. Flow chart of all identified studies on title/abstract level (title, description) [file 12939_2019_1098_MOESM2_ESM.docx]

**Flow chart of all identified studies on title/abstract level**

**Studies included in qualitative synthesis**

Diabetes: n = 5

Smoking: n = 8

Physical Activity: n = 3

Total: n = 16

**Full-text articles assessed for eligibility**

Diabetes: n = 6

Smoking: n = 11

Physical Activity: n = 4

Total: n = 21

**Records screened**

**Abstract/Title**

Diabetes: n = 141

Smoking: n = 80

Physical Activity: n = 263

Total: n = 484

**Records identified through PubMed**

Diabetes: n = 141

Smoking: n = 80

Physical activity: n = 263

Total: n = 484

**Full-text articles excluded**

Diabetes: n = 1

Not conducting interaction analysis:

n=1

Smoking: n = 3

Not mainly focusing on adult population:

n=3

Physical Activity: n = 1

Not mainly focusing on adult population:

n=1

Total: n = 5

**Records excluded
Abstract/Title excluded**

Diabetes: n = 135

Not referring to intersectionality:

n=133

Not conducting multivariable analysis:

n=2

Smoking: n = 69

Not referring to intersectionality:

n=59

Not conducting multivariable analysis:

n=10

Physical Activity: n = 259

Not referring to intersectionality:

n=246

Not conducting multivariable analysis:

n=13

Total: n = 463
