# Supplementary material for: Intersectionality-based quantitative health research and sex/gender sensitivity: a scoping review
Source: Int J Equity Health. 2019 Dec 21;18:199. doi: 10.1186/s12939-019-1098-8 (PMC6925460; doi:10.1186/s12939-019-1098-8)
Supplement: Supplementary file 3 — Additional file 3. Bibliography of included studies (title); List of references of included studies. [file 12939_2019_1098_MOESM3_ESM.docx]

**Bibliography of included studies**

1. Assari, S., Lankarani, M.M., Piette, J.D., & Aikens, J.E. (2017). Self-Rated Health and Glycemic Control in Type 2 Diabetes: Race by Gender Differences. *J Racial Ethn Health Disparities*, 5(4), 721-727.
2. Chesla, C.A., Kwan, C.M., Chun, K.M., & Stryker, L. (2014). Gender differences in factors related to diabetes management in Chinese American immigrants. *West J Nurs Res*, 36, 1074-1090.
3. Gagne, T., & Veenstra, G. (2017). Inequalities in Hypertension and Diabetes in Canada: Intersections between Racial Identity, Gender, and Income. *Ethn Dis*, 27, 371-378.
4. Salsberry, P.J., Corwin, E., & Reagan, P.B. (2007). A complex web of risks for metabolic syndrome: race/ethnicity, economics, and gender. *Am J Prev Med*, 33, 114-120.
5. Walmer, R., Huynh, J., Wenger, J., Ankers, E., Mantha, A.B., Ecker, J., et al. (2015). Mental health disorders subsequent to gestational diabetes mellitus differ by race/ethnicity. *Depress Anxiety*, 32, 774-782.
6. Aguirre, C.G., Bello, M.S., Andrabi, N., Pang, R.D., Hendricks, P.S., Bluthenthal, R.N., et al. (2015). Gender, Ethnicity, and Their Intersectionality in the Prediction of Smoking Outcome Expectancies in Regular Cigarette Smokers. *Behav Modif*, 40, 281-302.
7. Bilal, U., Beltran, P., Fernandez, E., Navas-Acien, A., Bolumar, F., & Franco, M. (2015). Gender equality and smoking: a theory-driven approach to smoking gender differences in Spain. *Tob Control*, 25, 295-300.
8. Cubbin, C., Soobader, M.J., & LeClere, F.B. (2010). The intersection of gender and race/ethnicity in smoking behaviors among menthol and non-menthol smokers in the United States. *Addiction*, 105 Suppl 1, 32-38.
9. Gaalema, D.E., Leventhal, A.M., Priest, J.S., & Higgins, S.T. (2018). Understanding individual differences in vulnerability to cigarette smoking is enhanced by attention to the intersection of common risk factors. *Prev Med*, 117, 38-42*.*
10. Klassen, A.C., Pankiewicz, A., Hsieh, S., Ward, A., & Curriero, F.C. (2015). The association of area-level social class and tobacco use with adverse breast cancer characteristics among white and black women: evidence from Maryland, 1992-2003*. Int J Health Geogr*, 14, 13.
11. Ortiz, K., Cuevas, A.G., Salloum, R., Lopez, N., & LaVeist-Ramos, T. (2018). Intra-Ethnic Racial Differences in Waterpipe Tobacco Smoking among Latinos? *Subst Use Misuse*, 1-10.
12. Pang, R.D., Bello, M.S., Liautaud, M.M., Weinberger, A.H., & Leventhal, A.M. (2018). Gender differences in negative affect during acute tobacco abstinence differ between African American and White adult cigarette smokers. *Nicotine Tob Res*.
13. Villanti, A.C., Gaalema, D.E., Tidey, J.W., Kurti, A.N., Sigmon, S.C., & Higgins, S.T. (2018). Co-occurring vulnerabilities and menthol use in U.S. young adult cigarette smokers: Findings from Wave 1 of the PATH Study, 2013-2014. *Prev Med*, 117, 43-51.
14. Abichahine, H., & Veenstra, G. (2016). Inter-categorical intersectionality and leisure-based physical activity in Canada. *Health Promot Int*, 32, 691-701.
15. Ray, R. (2017). Black people don't exercise in my neighborhood: Perceived racial composition and leisure-time physical activity among middle class blacks and whites. *Soc Sci Res*, 66, 42-57.
16. Wells, L., Nermo, M., & Ostberg, V. (2016). Physical Inactivity From Adolescence to Young Adulthood: The Relevance of Various Dimensions of Inequality in a Swedish Longitudinal Sample. *Health Educ Behav*, 44, 376-384.
