# Supplementary material for: Intersectionality-based quantitative health research and sex/gender sensitivity: a scoping review
Source: Int J Equity Health. 2019 Dec 21;18:199. doi: 10.1186/s12939-019-1098-8 (PMC6925460; doi:10.1186/s12939-019-1098-8)
Supplement: Supplementary file 4 — Additional file 4. Characteristics of selected papers (diabetes, smoking and physical activity) (title); This file shows the characteristics of the included studies in the field “diabetes”, “smoking” and “physical activity”. [file 12939_2019_1098_MOESM4_ESM.docx]

**Characteristics of selected papers (diabetes)**

|  | **Author**  **(year)** | **Title** | **Study location** | **Study population** | **Size of study population** | **Study design of data analysis** | **Inter- or**  **intra-inter-categorical approach** | **Main method.**  **approach** |
| --- | --- | --- | --- | --- | --- | --- | --- | --- |
| **a** | **Assari**  **et al. (2018)** | Self-rated health and glycemic control in type 2 diabetes: Race by gender differences. | US | Pooled data from urban health care system | N=287 | Cross-sectional | Inter | Linear  regression |
| **b** | **Chesla**  **et al. (2014)** | Gender differences in factors related to diabetes management in Chinese American immigrants | US | Convenience sample | N=178 | Single-cohort, delayed-treatment trial | Intra-inter: First generation  Chinese immigrants | Multilevel  linear  regression |
| **c** | **Gagne& Veenstra (2017)** | Inequalities in hypertension and diabetes in Canada: Intersections between racial identity, gender, and income | Canada | Pooled data from cycles of Canadian  Community Health Survey (CCHS) | N=613909 | Cross-sectional | Inter | Multilevel  logistic  regression |
| **d** | **Salsberry**  **et al. (2007)** | A complex web of risks for metabolic syndrome: race/ethnicity, economics, and gender | US | Pooled data from cycles of National Health and Nutrition Examination Survey (NHANES) | N=3049 | Cross-sectional | Inter | Logistic  regression |
| **e** | **Walmer**  **et al. (2015)** | Mental health disorders subsequent to gestational diabetes mellitus differ by race/ethnicity | US | Clinical cohort | N=15120 | Cohort  study | Intra-inter: Women | Logistic  Regression  with Generalized Estimating Equations |

**Characteristics of selected papers (smoking)**

|  | **Author**  **(year)** | **Title** | **Study location** | **Study population** | **Size of study population** | **Research design of data analysis** | **Inter- or**  **intra-inter-categorical approach** | **Main method.**  **approach** |
| --- | --- | --- | --- | --- | --- | --- | --- | --- |
| **f** | **Aguirre**  **et al. (2016)** | Gender, ethnicity, and their intersectionality in the prediction of smoking outcome expectancies in regular cigarette smokers | U.S. | Convenience sample | N=310 | Cross-sectional | Inter | Manova-based profile analysis |
| **g** | **Bilal**  **et al. (2016)** | Gender equality and smoking: A theory-driven approach to smoking gender differences in Spain | Spain | National Health Interview Survey (NHIS) | Not indicated | Ecological | Inter | Log-log linear regression |
| **h** | **Cubbin**  **et al. (2010)** | The intersection of gender and race/ethnicity in smoking behaviors among menthol and non-menthol smokers in the United States | U.S. | National Health Interview Survey (NHIS)  Cancer Control Supplement | N=21196 | Cross-sectional | Inter | Not indicated |
| **i** | **Gaalema**  **et al. (2018)** | Understanding individual differences in vulnerability to cigarette smoking is enhanced by attention to the intersection of common risk factors | U.S. | Pooled data from cycles of the US National Household Survey on Drug Use and Health (NSDUH) | N=127857 | Cross-sectional | Inter | CART-  Analysis |
| **j** | **Klassen**  **et al. (2015)** | The association of area-level social class and tobacco use with adverse breast cancer characteristics among white and black women: Evidence from Maryland, 1992-2003 | U.S. | Maryland Cancer  Registry  Census data  Consumer  Expenditure Survey (CEX) | N=50062 | Cross-sectional | Intra-inter:  Women | Multilevel  logistic  regression |
| **k** | **Ortiz**  **et al. (2018)** | Intra-Ethnic Racial Differences in Waterpipe Tobacco Smoking among Latinos? | U.S. | Pooled data from cycles of the National Adult  Tobacco Survey (NATS) | N=129649 | Cross-sectional | Inter | Log-Poisson regression |
| **l** | **Pang**  **et al. (2018)** | Gender Differences in Negative Affect During Acute Tobacco Abstinence Differ Between African American and White Adult Cigarette Smokers | U.S. | Convenience sample | N=789 | Cross-sectional | Inter | Linear regression |
| **m** | **Villanti**  **et al. (2018)** | Co-occurring vulnerabilities and menthol use in U.S. young adult cigarette smokers: Findings from Wave 1 of the PATH Study, 2013–2014 | U.S. | Population Assessment of Tobacco and Health Study (PATH) | N=15448 | Cross-sectional | Intra-inter:  Young  adults | Logistic regression  CART-  Analysis |

**Characteristics of selected papers (physical activity)**

|  | **Author**  **(year)** | **Title** | **Study location** | **Access to study population** | **Size of study population** | **Research design of data analysis** | **Inter- or**  **intra-inter-categorical approach** | **Main method.**  **approach** |
| --- | --- | --- | --- | --- | --- | --- | --- | --- |
| **n** | **Abichahine & Veenstra (2017)** | Inter-categorical intersectionality and leisure-based physical activity in Canada | Canada | Pooled data from cycles of the Canadian Community Health Survey (CCHS) | N=149574 | Cross-sectional | Inter | Multilevel  logistic  regression |
| **o** | **Ray**  **(2017)** | Black people don't exercise in my neighborhood: Perceived racial composition and leisure-time physical activity among middle class blacks and whites | U.S. | Existing  panel not representative for population | N=482 | Cross-sectional | Intra-inter:  Bachelor's degree or above and urban/suburban neighbourhoods | Logistic regression |
| **p** | **Wells**  **et al. (2017)** | Physical inactivity from adolescence to young adulthood: The relevance of various dimensions of inequality in a Swedish longitudinal sample | Sweden | Swedish  Level of Living Survey  Child-LNU | N=765 | Cross-  sectional | Intra-inter:  Young adults | Logistic regression |
